# Accuracies of bone resection, implant position, and limb alignment in robotic-arm-assisted total knee arthroplasty: a prospective single-centre study

**DOI:** 10.1186/s13018-022-02957-1

**Published:** 2022-01-29

**Authors:** Chenkai Li, Zian Zhang, Guanrong Wang, Chun Rong, Wanping Zhu, Xinzhe Lu, Yikai Liu, Haining Zhang

**Affiliations:** 1 Department of Joint Surgery, The Affiliated Hospital of Qingdao University, Qingdao, 266000 Shandong China; 2 Operating Room of Affiliated Hospital of Qingdao University, Qingdao, 266000 China

**Keywords:** Total knee arthroplasty, Robotic-arm-assisted technique, Bone resection, Implantation, Mechanical alignment

## Abstract

**Objective:**

This study assessed the accuracy of robotic-arm-assisted total knee arthroplasty (RATKA) for bone resection, component size prediction, implant placement, and limb alignment.

**Methods:**

This prospective cohort study included 36 patients. All procedures were performed by a single experienced surgeon, using an identical approach and implant designs. The MAKO RIO Robotic Interactive Orthopaedic Arm (Stryker, Mahwah, NJ, USA) system was used. The actual bone resection, implant placement, component size, and postoperative mechanical alignment were recorded, then compared with the preoperative plan.

**Results:**

The mean absolute differences from the plan for the distal (medial and lateral) and posterior (medial and lateral) femoral cuts were 0.39 mm (0.62), 0.49 mm (0.70), 0.62 mm (0.79), and 0.65 mm (0.81), respectively, with 0.57° (0.65) varus. The mean absolute differences in the medial and lateral tibial cuts were 0.56 mm (0.75) and 0.58 mm (0.76), with 0.48° (0.16) varus and 0.54° (0.25) anterior/posterior slope. Of 192 bone resections, 176 (91.7%) were within ≤ 1 mm of the preoperative plan. The accuracies of femoral and tibial component size prediction were 100% and 97.22%, respectively. The mean absolute difference in final limb coronal alignment was 0.92° (0.65). Of the alignments, 18 (75.0%) were within ≤ 1.00° of the plan, and 100% were within ≤ 3.00° of the plan.

**Conclusion:**

RATKA could accurately predict the component size and execute a preoperative plan to achieve precise bone resection, and implant placement, thereby reducing alignment outliers.

## Background

The precision of implantation, mechanical alignment, and soft tissue balance are important for the success of total knee arthroplasty (TKA) [1]. It had been reported that at 25 years follow-up, the implant survivorship of TKA was appropriately 82% [2]. Deviation during the operation, such as malposition and imbalance, will affect implant survival [3]. Malposition will increase wear and result in poor clinical outcomes, which may lead to early revision [4]. Imbalance is a risk factor for pain, instability, and aseptic loosening, which may eventually decrease patient satisfaction [5]. It may be difficult for surgeons to consistently achieve the ideal implantation and alignment of components; surgical errors are the major cause of TKA failure.

Robotic-arm-assisted surgery in orthopaedics began in the 1980s; it was developed to enhance the accuracies of bone resection, implant selection and placement, and alignment, thereby improving patient clinical function and long-term implant survival [6]. Robotic-arm-assisted total knee arthroplasty (RATKA) has gathered momentum as a method to improve the accuracies of bone resection and implant position, thus reducing limb alignment outliers [7]. In these systems, robotic-arm-assisted surgeons precisely machine the bones, using preoperative computed tomography-based planning and haptic feedback. Bäthis et al. [8] confirmed the accuracy of RATKA by analysing bone resection in 50 cases. Cheng et al. [9] demonstrated that RATKA improved the precision of implant position and mechanical alignment. Besides, Mason et al. [10] reported that 91% of patients achieved mechanical alignment deviation within ± 3°. This study evaluated the accuracy and precision of RATKA for predicting component size and achieving the preoperatively planned bone resection, implant position, and final limb coronal mechanical alignment.

## Materials and methods

### Patient selection

Between June 2021 and July 2021, 36 patients with symptomatic end-stage knee osteoarthritis underwent primary RATKA at the same centre. All procedures were performed by an experienced surgeon trained in RATKA. Patients with knee osteoarthritis undergoing primary TKA were recruited for the study. Exclusion criteria were: varus or valgus deformity > 20°; neurological disorders or inflammatory arthropathies; and previous leg surgery. The study was assessed by the hospital review board which waived the need for further ethics approval.

### Surgical technique

The MAKO RIO Robotic Interactive Orthopaedic Arm (Stryker, Mahwah, NJ, USA) system was used. A three-dimensional model of the patient’s bony anatomy was used to plan the optimal bone resection, implant positioning, and limb alignment. Femoral and tibial registration pins were inserted for intraoperative dynamic tracking; they were used to assess range of movement and mechanical alignment. A conventional medial parapatellar approach was used in all patients. After registration and adjustments of the preoperative plan, the robotic-arm-assisted surgeon performs femoral and tibial osteotomies within a virtual haptic boundary. In the coronal plane, femoral and tibial varus/valgus were both set to 0°. In the sagittal plane, the femoral component flexion was initially set to 4°, while the tibial slope was set to 0°. The surgeon could adjust these settings as necessary based on an intraoperative assessment of the flexion gap and range of movement. All patients used fully cemented implantation (Triathlon Posterior Stabilisation Total Knee System, Stryker Orthopaedics, Mahwah, NJ, USA).

### Data collection

The preoperative plan included bone resection, component placement, and mechanical alignment targets. Patients were assessed intraoperatively or before discharge in terms of bone resection and prediction accuracy; knee X-rays and a weight-bearing scanogram were obtained. (1) Bone resection included the distal femur (medial and lateral), posterior femur (medial and lateral), and tibia (medial and lateral) cuts, without considering cartilage. Both the planned and actual bone resections were recorded. The actual bone resection was measured intraoperatively using Vernier calipers after the osteotomy; the percentage of bone resection ≤ 1 mm from the preoperative plan was recorded. (2) The accuracies of predicting the femoral and tibial component sizes were checked. (3) Knee X-rays were used to assess the implant position, including the femoral and tibial component varus/valgus positions from the mechanical axes, as well as tibial component posterior slope. (4) Weight-bearing scanograms were used to evaluate mechanical alignment. The original targeted alignment was neutral alignment, but surgeon might adjust it according to details of each patient. The outcomes were defined based on the degree of deviation as “excellent” (0–1.99°), “acceptable” (2–2.99°), and an “outlier” (≥ 3°), compared with the preoperative planned.

### Statistical analysis

IBM SPSS Statistics, version 24.0, was used to analyse the data. Continuous variables are expressed using means and standard deviations; categorical variables are expressed using frequencies and percentages. Accuracy was defined as the root mean square (RMS) of the measurements. Precision was defined as the standard deviation of measurements.

## Results

This prospective study enrolled 36 patients (mean age, 67.6 ± 6.1 years; 83.33% women; 94.44% varus). Eleven patients (30.56%) had surgery on their left knees.

**Fig. 1:**
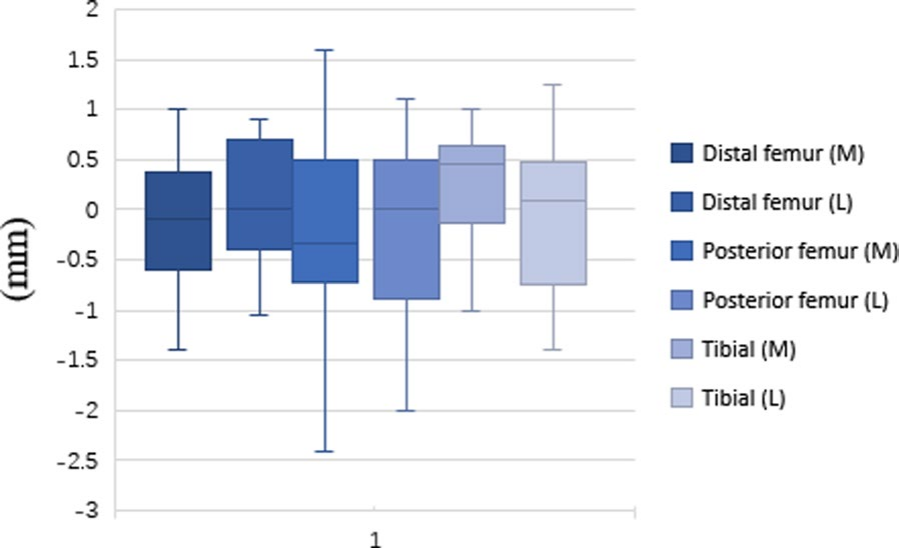
Box–whisker plots of the bone resections including maximum, minimum, median, and interquartile range values. Positive values indicating more bone was resected than planned. *M* medial; *L* lateral

The mean absolute differences from the plan for distal (medial and lateral) and posterior (medial and lateral) femoral cuts were 0.39 mm (0.62), 0.49 mm (0.70), 0.62 mm (0.79), and 0.65 mm (0.81), respectively, with 0.57° (0.65) varus. The mean absolute difference in the medial and lateral tibial cuts was 0.56 mm (0.75) and 0.58 mm (0.76), with 0.48° (0.16) varus and 0.54° (0.25) anterior/posterior slope (Table 1). Of 192 of bone resections, 176 (91.67%) were ≤ 1 mm from the preoperative plan. Figure 1 summarises the bone resections. The accuracies of femoral and tibial component size prediction were 100% and 97.22%, respectively.

**Table 1:** Summary of bone resection compared to the plan

Bone resection	Mean (mm)	RMS (mm)	Max. error (mm)	≤ 1 mm (%)
Distal femur cut				
Deep (medial)	− 0.08 (0.61)	0.39 (0.62)	2.00	93.75
Deep (lateral)	− 0.03 (0.70)	0.49 (0.70)	2.40	93.75
Posterior femur cut				
Deep (medial)	− 0.18 (0.77)	0.62 (0.79)	2.40	87.50
Deep (lateral)	− 0.17 (0.79)	0.65 (0.81)	2.00	90.63
Tibial cut				
Deep (medial)	0.38 (0.66)	0.56 (0.75)	1.90	93.75
Deep (lateral)	− 0.13 (0.75)	0.58 (0.76)	1.40	84.38

*RMS* root mean square

**Fig. 2:**
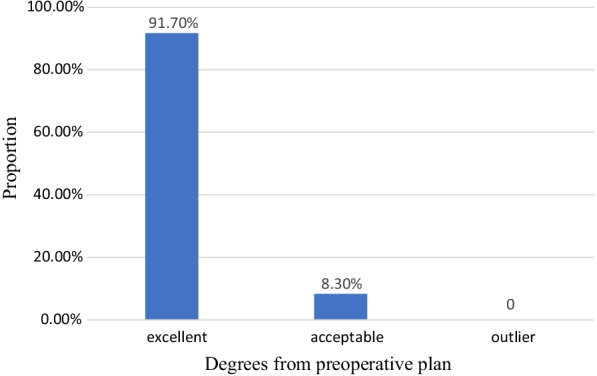
Proportion of knees with final mechanical alignment from the preoperative plan

The mean absolute difference in the final limb coronal alignment was 0.92° (0.65): 27 alignments (75.00%) were ≤ 1.00° of the plan, and 100% were within ≤ 3.00° of the plan. Overall, 33 knees were classified as “excellent”, 3 as “acceptable”, and 0 as “outlier” (Fig. 2).

## Discussion

In this prospective study, RATKA exhibited high accuracies for bone resection, implantation, and correction of limb alignment. The robotic arm followed the preoperative plan accurately. Further studies should enrol multiple surgeons and include more cases to analyse the interaction between surgical accuracy and clinical outcome.

Because the sawblade action was confined to the preoperative plan in RATKA, it helped to improve the accuracy and precision of femoral and tibial bone resection. Hampp et al. [11] compared the error of bone resection between RATKA and conventional TKA in six cadavers; compared with conventional TKA, RATKA enhanced the precision and accuracy in achieving preplanned implantation. In 45 consecutive RATKAs, Sires et al. [12] found that the accuracy of RATKA in achieving planned bone resection was high, such that 99 of 105 bone resections were within 1 mm of the preoperative plan. Bäthis et al. [8] also confirmed the accuracy of bone resection in RATKA; the deviations of distal femur and tibial resection were 0.6 mm (0.4) and 0.7 mm (0.5), respectively. Overall, robotic-arm-assisted techniques could accurately execute the preoperative bone resection plan and achieve the ideal flexion–extension gap balance, which are important for mechanical alignment and ligament tension. Thus, it is helpful for surgeons to introduce robotic-arm-assisted techniques into their routine treatment.

Deviation of implant placement is a risk factor for aseptic loosening [5]. The achievement of ideal, patient-specific component placement requires precise, accurate implant positioning. Comparing 143 consecutive RATKA cases and 151 consecutive conventional manual TKA cases, Thilak et al. [13] found that RATKA achieved accurate preoperatively planned implant positioning. In a prospective study, Mahoney et al. [14] found that compared with conventional manual TKA, the radiographic parameters of component positioning were improved in the RATKA cohort; accuracy improvements were greatest in femoral component rotation, tibial component alignment, and slope. Using computed tomography scans to compare the accuracy in 10 RATKA cases and 10 conventional TKA cases, Moon et al. [15] demonstrated that RATKA achieved greater accuracy in implantation and alignment; it also more accurately restored the posterior condylar offset ratio, Insall–Salvati ratio, and native joint line. They found a mean flexion/extension deviation of 1.4° and mean varus/valgus deviation of 0.6° for the femoral cut; the varus/valgus alignment showed a mean deviation of 0.5° for the proximal tibial cut. However, Kim et al. [16] and Cip et al. [17] found no difference between RATKA and conventional TKA groups in terms of implantation accuracy with both coronal and sagittal radiological assessment. We also found that a robotic-arm-assisted technique accurately predicted the component size. Notably, Marchand et al. [18] reported that the accuracy of RATKA in predicting the femoral and tibial component was 98%.

Malalignment in the sagittal and coronal planes is associated with instability, aseptic loosening, and pain [19]. Postoperative alignment is influenced by the extent of preoperative varus deformity [20]. Although there remains no consensus regarding whether component alignment within ± 3° of the neutral mechanical axis is related to longer survival rates and better long-term function, Parratte et al. [21] demonstrated that the neutral mechanical axis should be recognised as the gold standard until the biomechanical relationship of component alignment in the axis and sagittal and coronal planes is better understood. Studies have demonstrated that the error range of the conventional manual technique is 13–38% when using an intramedullary femoral and extramedullary tibial jig [22, 23]. The errors could be caused by variations in native bony anatomy or the use of oscillating saws for bone preparation. Several studies have reported RATKA can accurately restore a mechanical alignment close to 180° and minimise alignment outliers. Liow et al. [24] demonstrated that RATKA improved the accuracies of component orientation and mechanical alignment. In a prospective study of 50 RATKA cases versus 50 conventional TKA cases, RATKA accurately performed the preoperative plan and reduced the number of outliers (> 3°) [7]. Another study confirmed the accuracy of RATKA for correcting mechanical alignment, such that 78.13% of alignments were within ≤ 1°of the plan and 100% were within ≤ 3° of the plan [12]. However, Spencer et al. [25] reported that there is no difference in overall knee alignment between RATKA and conventional TKA. In addition, Song et al. [23] and Park et al. [26] found no significant differences in alignment of the limb axis between the two techniques.

This study had some limitations. First, the sample size was small and future studies involving larger numbers are necessary. Second, because the radiographic data were obtained early in the postoperative period, pain might have negatively impacted the accuracy of radiological measurements from standard radiographs. Furthermore, there is measurement error in bone resection using Vernier calipers, although the error is minimal. Finally, the patients’ reported outcomes were not collected. Thus, a future study should analyse the association between the accuracy of RATKA and patients’ reported outcomes.

## Conclusion

RATKA accurately predicted the component size and executed the preoperative plan to achieve precise bone resection, and implant placement, thereby reducing alignment outliers. Future studies should analyse whether this robust accuracy is associated with better functional outcomes and greater implant longevity.

## Data Availability

The final dataset will be available from the corresponding author.

## Abbreviations

RATKA: Robotic-arm-assisted total knee arthroplasty
TKA: Total knee arthroplasty

## References

[1] Jakopec M, Harris SJ, Rodriguez y Baena F (2001). The first clinical application of a “hands-on” robotic knee surgery system. Comput Aided Surg.

[2] Haddad FS (2017). What is the optimal level of expectation?. Bone Jt J.

[3] Vertullo CJ, Graves SE, Cuthbert AR (2019). The effect of surgeon preference for selective patellar resurfacing on revision risk in total knee replacement: an instrumental variable analysis of 136,116 procedures from the Australian Orthopaedic Association National Joint Replacement Registry. J Bone Jt Surg Am.

[4] Lotke PA, Ecker ML (1977). Influence of positioning of prosthesis in total knee replacement. J Bone Jt Surg Am.

[5] Ritter MA, Davis KE, Meding JB (2011). The effect of alignment and BMI on failure of total knee replacement. J Bone Jt Surg Am.

[6] Paul HA, Bargar WL, Mittlestadt B (1992). Development of a surgical robot for cementless total hip arthroplasty. Clin Orthop Relat Res.

[7] Kayani B, Konan S, Ayuob A (2019). Robotic technology in total knee arthroplasty: a systematic review. EFORT Open Rev.

[8] Bathis H, Perlick L, Tingart M (2005). Intraoperative cutting errors in total knee arthroplasty. Arch Orthop Trauma Surg.

[9] Cheng T, Zhao S, Peng X (2012). Does computer-assisted surgery improve postoperative leg alignment and implant positioning following total knee arthroplasty? A meta-analysis of randomized controlled trials?. Knee Surg Sports Traumatol Arthrosc.

[10] Mason JB, Fehring TK, Estok R (2007). Meta-analysis of alignment outcomes in computer-assisted total knee arthroplasty surgery. J Arthroplasty.

[11] Hampp EL, Chughtai M, Scholl LY (2019). Robotic-arm assisted total knee arthroplasty demonstrated greater accuracy and precision to plan compared with manual techniques. J Knee Surg.

[12] Sires JD, Craik JD, Wilson CJ (2021). Accuracy of bone resection in MAKO total knee robotic-assisted surgery. J Knee Surg.

[13] Thilak J, Babu BC, Thadi M (2021). Accuracy in the execution of pre-operative plan for limb alignment and implant positioning in robotic-arm assisted total knee arthroplasty and manual total knee arthroplasty: a prospective observational study. Indian J Orthop.

[14] Mahoney O, Kinsey T, Sodhi N (2020). Improved component placement accuracy with robotic-arm assisted total knee arthroplasty. J Knee Surg.

[15] Moon YW, Ha CW, Do KH (2012). Comparison of robot-assisted and conventional total knee arthroplasty: a controlled cadaver study using multiparameter quantitative three-dimensional CT assessment of alignment. Comput Aided Surg.

[16] Kim YH, Park JW, Kim JS (2012). Computer-navigated versus conventional total knee arthroplasty a prospective randomized trial. J Bone Jt Surg Am.

[17] Cip J, Obwegeser F, Benesch T (2018). Twelve-year follow-up of navigated computer-assisted versus conventional total knee arthroplasty: a prospective randomized comparative trial. J Arthroplasty.

[18] Marchand RC, Sodhi N, Bhowmik-Stoker M (2019). Does the robotic arm and preoperative CT planning help with 3D intraoperative total knee arthroplasty planning?. J Knee Surg.

[19] Meijer MF, Reininga IH, Boerboom AL (2014). Does imageless computer-assisted TKA lead to improved rotational alignment or fewer outliers? A systematic review. Clin Orthop Relat Res.

[20] Bae DK, Song SJ, Yoon KH (2012). Comparative study of tibial posterior slope angle following cruciate-retaining total knee arthroplasty using one of three implants. Int Orthop.

[21] Parratte S, Pagnano MW, Trousdale RT (2010). Effect of postoperative mechanical axis alignment on the fifteen-year survival of modern, cemented total knee replacements. J Bone Jt Surg Am.

[22] Song EK, Seon JK, Park SJ (2011). Simultaneous bilateral total knee arthroplasty with robotic and conventional techniques: a prospective, randomized study. Knee Surg Sports Traumatol Arthrosc.

[23] Song EK, Seon JK, Yim JH (2013). Robotic-assisted TKA reduces postoperative alignment outliers and improves gap balance compared to conventional TKA. Clin Orthop Relat Res.

[24] Liow MH, Xia Z, Wong MK (2014). Robot-assisted total knee arthroplasty accurately restores the joint line and mechanical axis. A prospective randomised study. J Arthroplasty.

[25] Spencer JM, Chauhan SK, Sloan K (2007). Computer navigation versus conventional total knee replacement: no difference in functional results at two years. J Bone Jt Surg Br.

[26] Park SE, Lee CT (2007). Comparison of robotic-assisted and conventional manual implantation of a primary total knee arthroplasty. J Arthroplasty.

